# Depth of Invasion Predicts Vocal Fold Motility and Muscle Invasion in Glottic Carcinoma

**DOI:** 10.1002/lary.70784

**Published:** 2026-07-31

**Authors:** Elisa Bellini, Claudio Sampieri, Francesco Chu, Marta Filauro, Monica Feltri, Maurizio Boggio, Paolo Nozza, Stefano Filippo Zorzi, Francesco Bandi, Simone Caprioli, Edoardo Nizzi, Francesca Ruju, Giuseppe Petralia, Francesco Cicchetti, Emanuele Zuccari, Ionela Aurora Bercea, Giuseppe Cittadini, Marco Ravanelli, Cesare Piazza, Mohssen Ansarin, Snehal Patel, Giorgio Peretti, Filippo Marchi

**Affiliations:** ^1^ Unit of Otorhinolaryngology‐Head and Neck Surgery IRCCS Azienda Ospedaliera Metropolitana Genoa Italy; ^2^ Department of Surgical Sciences and Integrated Diagnostics (DISC) University of Genoa Genoa Italy; ^3^ Department of Otorhinolaryngology and Head and Neck Cancer Unit Hospital Clínic Barcelona Spain; ^4^ Head and Neck Cancer Unit Hospital Clinic Barcelona Spain; ^5^ Department of Otolaryngology and Head and Neck Surgery European Institute of Oncology (IEO), IRCCS Milan Italy; ^6^ U.O. Anatomia Patologica Ospedaliera, IRCCS Azienda Ospedaliera Metropolitana, Plesso: Ospedale Policlinico San Martino Genova Italy; ^7^ Unit of General and Interventional Radiology IRCCS Ospedale Policlinico San Martino Genoa Italy; ^8^ Department of Health Sciences (DISSAL), Diagnostic Imaging and Radiotherapy University of Genoa Genoa Italy; ^9^ Division of Radiology European Institute of Oncology (IEO), IRCCS Milan Italy; ^10^ Postgraduate School in Radiodiagnostics University of Milan Milan Italy; ^11^ Clinic of Radiology and Medical Imaging Fundeni Clinical Institute Bucharest Romania; ^12^ Department of Medical and Surgical Specialties, Radiological Sciences, and Public Health University of Brescia Brescia Italy; ^13^ Unit of Otorhinolaryngology – Head and Neck Surgery ASST Spedali Civili of Brescia Brescia Italy; ^14^ Department of Surgical and Medical Specialties, Radiological Sciences, and Public Health (DSMC) University of Brescia, School of Medicine Brescia Italy; ^15^ Department of Surgery Memorial Sloan Kettering Cancer Center New York New York USA

**Keywords:** depth of invasion, laryngeal cancer, laryngeal motility, larynx carcinoma, staging system

## Abstract

**Objective:**

Vocal fold motility guides staging of glottic squamous cell carcinoma (SCC) but is subjective and operator dependent. Because impaired mobility reflects tumor depth of invasion (DOI) into the vocal muscle and paraglottic space, objective DOI measurement may better capture aggressiveness and functional impairment. This two‐stage study assessed radiologic–pathologic agreement of DOI, derived pathologic DOI (pDOI) thresholds for altered motility and deep muscle infiltration, and externally validated these thresholds, aiming to support a more reproducible staging surrogate.

**Methods:**

Surgically treated glottic SCC from Ospedale Policlinico San Martino (Genoa, Italy) formed the derivation cohort; external validation was performed at the European Institute of Oncology (Milan, Italy). Outcomes included correlation/agreement between radiologic DOI (rDOI) and pDOI, and between pathologic depth of vocal muscle invasion (pDMI). Spearman's *ρ*, Bland–Altman analysis, and Lin's concordance correlation coefficient (CCC) assessed agreement. ROC analysis (Youden index), likelihood ratios, and Fagan nomograms evaluated diagnostic performance and clinically meaningful thresholds.

**Results:**

In 262 derivation cases, rDOI strongly correlated with pDOI (*ρ* = 0.75; CCC = 0.824). pDOI predicted altered vocal fold motility (AUC 0.725; cutoff 2.1 mm) and accurately predicted muscle invasion (AUC 0.94; cutoff 2.2 mm), whereas pDMI showed moderate prediction of motility (AUC 0.69). In 38 validation cases, correlation remained significant (*ρ* = 0.56; CCC = 0.56), and Genoa‐derived DOI cutoffs were reproducible (sensitivity 85%–94%, specificity 76%–83%).

**Conclusions:**

DOI provides a robust, objective surrogate of functional impairment and muscle infiltration in glottic SCC, with externally validated thresholds that may complement or replace subjective mobility assessment in future staging algorithms.

**Level of Evidence:**

3.

## Graphical abstract

To determine whether depth of invasion (DOI) provides an objective surrogate for vocal fold motility and vocal muscle invasion in glottic squamous cell carcinoma. *Methods and Results*: In derivation and external validation cohorts, radiologic DOI strongly correlated with pathologic DOI and accurately predicted altered vocal fold motility and muscle invasion using reproducible thresholds of approximately 2–2.2 mm. *Conclusion*: DOI is a reliable, reproducible imaging biomarker that may complement or replace subjective vocal fold mobility assessment in future glottic cancer staging.

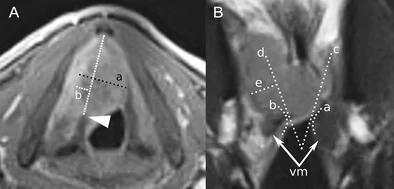

## 1 Introduction

Vocal fold motility (VFM) has long been considered a key element in the staging, prognosis, and treatment selection of laryngeal squamous cell carcinoma (SCC) [1, 2]. Despite its clinical significance, the assessment of VFM remains an inherently subjective process [3] susceptible to inconsistencies and interobserver variability. A review of the AJCC TNM staging manual reveals that no other solid tumor in the human body is staged on the basis of a subjective parameter, such as the assessment of VFM in laryngeal SCC [1, 2]. Moreover, staging of laryngeal tumors has remained largely unchanged from 1977 to the present day, where impaired VFM (cT2) and fixation (cT3) remain the two pivotal criteria [1, 2, 3, 4]. This lack of evolution directly contravenes the principles articulated by the founding editors of the Cancer Staging Manual, who asserted that the system was “intended to provide a means by which information can be readily communicated to others and serve as a factor in prognostic assessment,” and that “as new information becomes available regarding etiology and emerging diagnostic and therapeutic modalities, the classification and staging of cancer will evolve accordingly” [4].

Current literature reports variability in the evaluation of VFM, even in experienced raters [3]. These limitations are most evident at the T1–T2 and T2–T3 boundaries. Tumors may be classified as pT2 based only on preoperative assessment of impaired vocal fold mobility (VFM), although distinguishing hypomobility with preserved arytenoid movement from impaired mobility with arytenoid fixation depends on subtle, subjective endoscopic findings. This underscores the ambiguity of the classification: a cT2 lesion may correspond to a pT1, or vice versa, depending on how endoscopic findings are interpreted and translated into the final pathologic assessment. Such variability introduces inconsistency in both the prognostic stratification of surgically treated cases and the evaluation of patients managed with non‐surgical approaches. In this context, modern imaging supports a more objective staging approach, with depth of invasion (DOI) emerging as a promising prognostic marker of tumor aggressiveness [5].

The aims of this study were two‐fold. First, in a single‐institution derivation cohort from Genoa, we quantified the correlation and agreement between radiologic (rDOI) and pathologic depth of invasion (pDOI) and identified pDOI and pathologic depth of muscle infiltration (pDMI) thresholds linked to altered VFM. Second, we externally validated these thresholds in an independent cohort from another institution. We sought to establish DOI as an objective and clinically reliable parameter that can complement the inherently subjective assessment of VFM in glottic SCC staging.

## 2 Materials and Methods

### 2.1 Study Design and Ethical Approval

This was an observational retrospective study consisting of a derivation cohort of patients with glottic SCC, treated at IRCCS Ospedale Policlinico San Martino, University of Genoa, Italy, between 2015 and 2024, and an external validation cohort from the European Institute of Oncology (IEO), IRCCS, Milan, Italy [6].

Primary outcomes included the evaluation of rDOI, pDOI and pDMI. Eligible patients were adults (≥ 18 years) with primary glottic SCC treated with curative‐intent surgery with complete medical records and no distant metastases at presentation. Exclusion criteria comprised prior (chemo)radiotherapy [(C)RT] to the head and neck, previous head and neck cancer, and any previous laryngeal surgery. All patients underwent preoperative high‐definition videolaryngoscopy (HD‐VLS) that was recorded. Preoperative computed tomography (CT) or magnetic resonance imaging (MRI) was performed to evaluate deep tumor extension and paraglottic space (PGS) invasion according to published criteria [7, 8]. In the Genoa derivation cohort, patients were treated with transoral laser microsurgery (TOLMS), open partial laryngectomy (OPHL), or total laryngectomy (TL), depending on tumor extent, VFM, and patient‐related factors; in the IEO validation cohort, all patients were uniformly treated with TOLMS. The study was approved by the local ethics committees of both institutions (Genoa: ID LAR_MULTI; IEO: UID 4304). Reporting followed the STROBE guidelines for cohort studies [9].

### 2.2 Assessment of VFM, Radiologic, and Pathologic Protocols

In the Genoa derivation cohort, preoperative VFM was independently assessed by 3 raters on recorded preoperative videos and classified as preserved, altered, or not assessable; discordant cases were reviewed by a fourth consultant, and consensus was reached when at least 3 raters agreed.

rDOI was measured as the perpendicular distance from the reconstructed plane of the normal mucosa to the deepest point of tumor extension, excluding exophytic components and including ulcerated parts (Figure 1). CT scans were acquired in the venous phase following intravenous iodinated contrast, with 0.7 mm slice thickness, during quiet respiration and the neck in neutral resting position. MRI was performed with surface coils, using T1‐ and T2‐weighted sequences, diffusion‐weighted imaging, and post‐gadolinium fat‐saturated 3D T1‐weighted gradient‐echo sequences. Slice thickness was 3 mm for the pre‐contrast sequences and 0.8 mm for the post‐contrast images.

**FIGURE 1:**
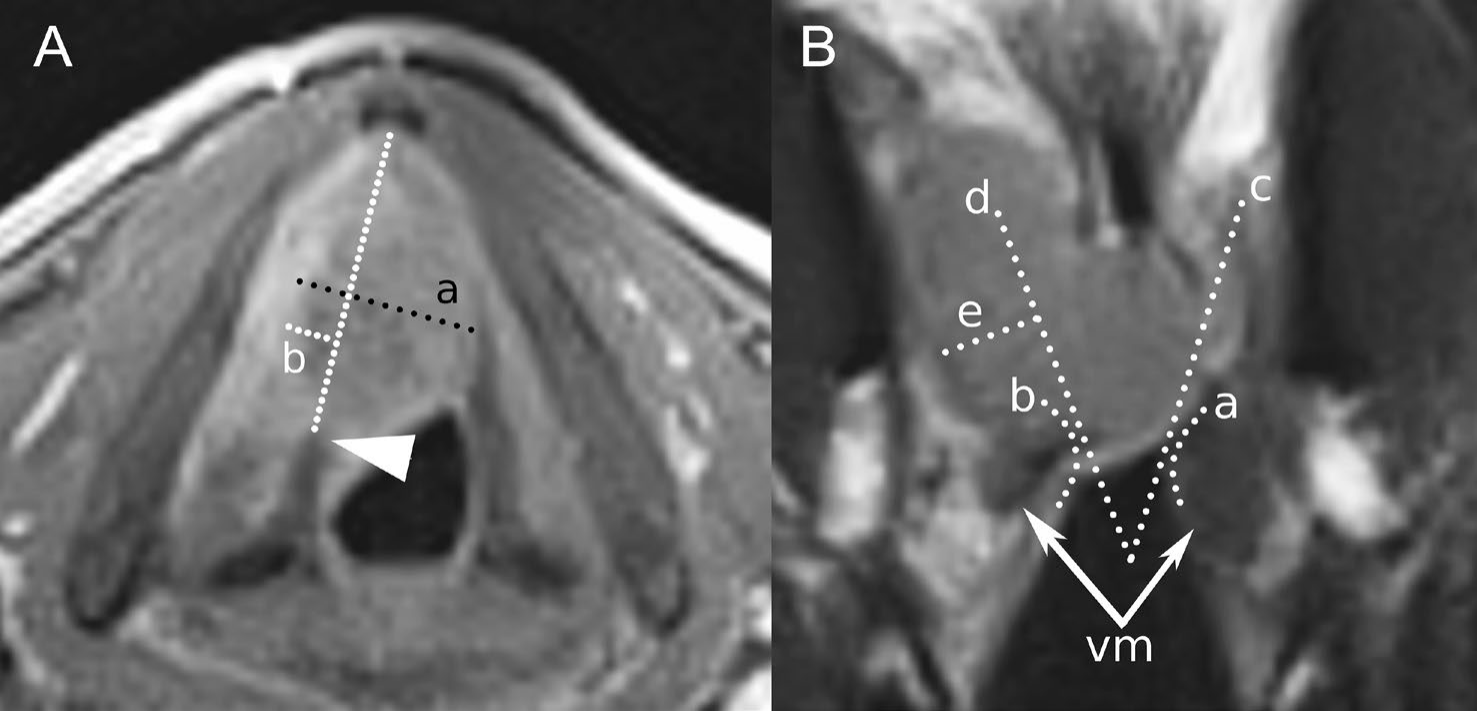
Examples of radiologic depth of invasion (rDOI) assessment on contrast‐enhanced magnetic resonance. (A) Post‐contrast T1‐weighted axial image. Stepwise measurement of radiologic depth of invasion (rDOI): Draw a reference line from the vocal process of the arytenoid (arrowhead) to the anterior commissure, then project a perpendicular segment from this line to the deepest tumor margin; segment (b) corresponds to rDOI. Note the distinction between rDOI and maximum tumor thickness (a). (B) T2‐weighted coronal image of the same patient. On the coronal plane, rDOI assessment may be more challenging because the curved anatomy and normal anatomic landmarks can be partially obscured by tumor. Stepwise measurement of rDOI: Identify the vocalis muscle and true vocal folds, and delineate their profiles on the healthy side (a) and the pathologic side (b); when the affected‐side contour is indistinct, mirroring the contralateral healthy profile can be used as a pragmatic reference. Draw tangential lines (c, d) and then project a perpendicular segment from line (d) to the deepest tumor margin; segment (e) corresponds to rDOI.

pDOI was defined analogously to the rDOI but on hematoxylin–eosin pathology sections, while pDMI was measured at 10× magnification using a micrometric lens by projecting a perpendicular line from the deepest layer of the vocal ligament to the deepest infiltrating tumor focus according to a standardized protocol (Figure 2).

**FIGURE 2:**
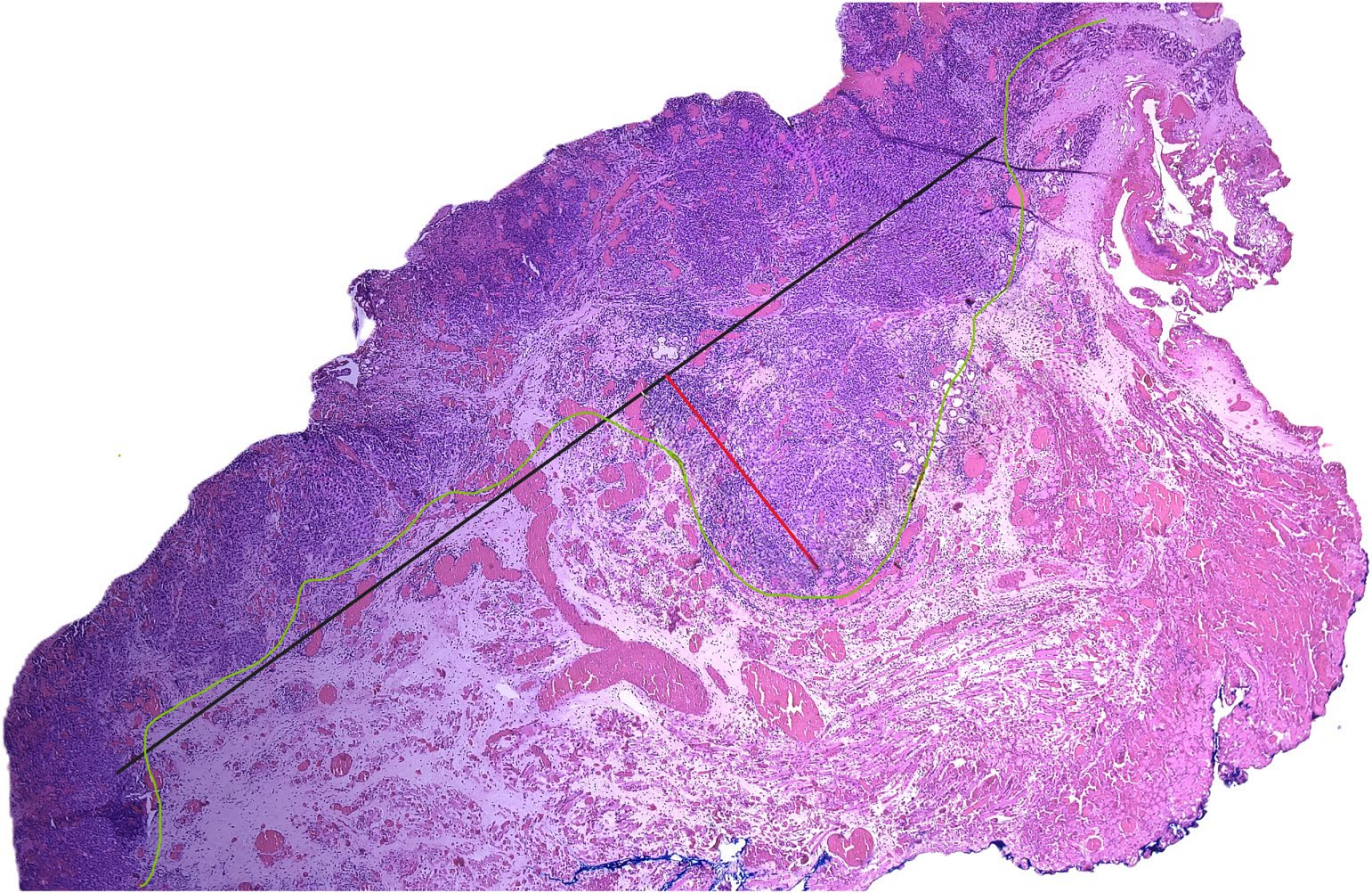
Hematoxylin–eosin sections of the vocal cord at 5×, showing a squamous cell carcinoma infiltrating the vocal muscle. Black lines indicate the plane parallel to the muscle surface at the first visible muscle fibers. Pathologic depth of vocal muscle invasion (pDMI) is measured as the length of the red perpendicular line extending to the deepest infiltrating tumor front (outlined in green). [Color figure can be viewed in the online issue, which is available at www.laryngoscope.com]

In the IEO validation cohort, endoscopic VFM was independently reviewed by 2 consultants, with disagreements resolved by a third. pDOI was re‐extracted for 197 eligible cases, and all available scans were re‐evaluated by 2 expert head and neck radiologists for rDOI re‐assessment.

### 2.3 Statistical Analysis

All data processing and statistical analyses were performed using Microsoft Excel, Jamovi (2.6.23), and RStudio (version 2025.09.1+401). Continuous variables were summarized as medians with interquartile ranges (IQR), while categorical variables were reported as absolute counts and percentages. Interobserver agreement was quantified using Fleiss' kappa for the two cohorts. Because DOI and DMI showed marked non‐normality, non‐parametric correlation methods were applied. Radiologic–pathologic association was evaluated using Spearman's *ρ*, and agreement was assessed through Bland–Altman plots and Lin's concordance correlation coefficient (CCC). Diagnostic performance of rDOI, pDOI, and pDMI was quantified using Receiver Operating Characteristic (ROC) curve analysis, reporting the Areas Under the Curve (AUCs), optimal cut‐offs based on the Youden index, and corresponding sensitivity, specificity, and likelihood ratios. Fagan nomograms were used to illustrate the clinical impact of Genoa‐derived thresholds across different pre‐test probabilities.

## 3 Results

In the derivation cohort, a total of 262 patients with glottic SCC were included, with a mean age of 69.3 years (range, 35–94). Detailed clinical and demographic characteristics are presented in Table 1.

**TABLE 1:** Baseline clinical, radiologic, and pathologic characteristics of the derivation cohort (*N* = 262).

Characteristic	Value
Gender	
Female	35 (13%)
Male	227 (87%)
Age, years	69 (62–77)
Smoking	209 (81%)
Alcohol use	91 (36%)
Side	
Unilateral	187 (71%)
Bilateral	75 (29%)
Altered vocal motility	138 (53%)
Pathologic T category	
pT1	174 (66%)
pT2	15 (5.7%)
pT3	60 (23%)
pT4	13 (5%)
Type of surgery	
Transoral laser microsurgery	222 (85%)
Total laryngectomy	21 (8%)
Open partial laryngectomy	19 (7.3%)
Radiologic DOI (mm)	4 (1.7–7)^a^
Pathologic DOI (mm)	2 (0.8–4)^a^
Pathologic DMI (mm)	0 (0–1.2)^a^

*Note:* Continuous variables are presented as median values with interquartile ranges (IQR); categorical variables are shown as absolute counts with percentages.

Abbreviations: DMI, depth of vocal muscle invasion; DOI, depth of infiltration.

^a^ Number of patients with available measurements: Radiologic DOI = 94; pathologic DOI = 212 patients; radiologic DMI = 91; pathologic DMI = 212 patients.

VFM was assessed as normal in 47.3% of patients and impaired in 52.7%. Inter‐observer agreement was reached in 71% of cases (*κ* = 0.685). The remaining discordant cases were subsequently reviewed by a fourth consultant, resulting in an overall agreement rate of 86.3% (*κ* = 0.675).

rDOI measurements were available for 55.9% patients who underwent preoperative imaging, with a median value of 4 mm (IQR, 1.7–7 mm). pDOI and pDMI were available for 80.9% of surgically treated patients and displayed markedly skewed distributions. pDOI demonstrated a median of 2 mm (IQR, 0.8–4 mm), consistent with the predominance of early‐stage i glottic SCC. In contrast, pDMI exhibited a heavily zero‐inflated distribution. More than 50% of patients showed no vocal muscle infiltration, resulting in a median of 0 mm and a long right‐sided tail extending up to 7 mm, an anatomically expected pattern given the high pT1 prevalence in the present cohort.

### 3.1 Correlation Between rDOI‐pDOI‐pDMI

Spearman analysis demonstrated a strong association between rDOI and pDOI (*ρ* = 0.75, *p* < 0.001) (Figure 3), confirming that radiologic measurements generally follow the same trend as pathological depth. Bland–Altman analysis showed a mean difference between the two measurements of 0.3 mm, with 95% limits of agreement from −4.6 to 5.2 mm. Consistently, Lin's CCC was 0.8 (95% CI: 0.7–0.8), indicating good agreement supporting rDOI as reliable surrogate for true depth of invasion.

**FIGURE 3:**
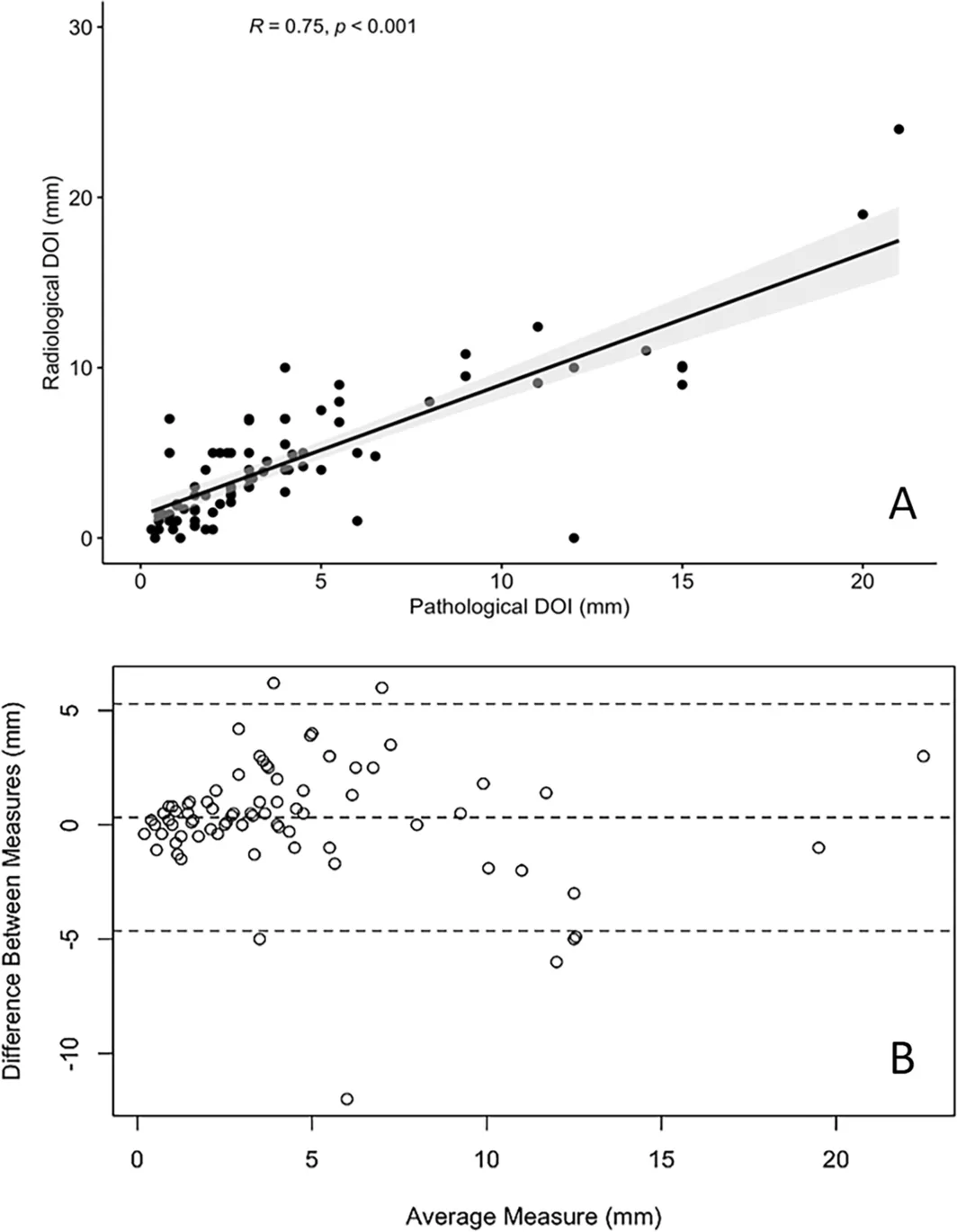
Correlation and agreement between radiologic depth of invasion (rDOI) and pathologic depth of invasion (pDOI) in the derivation cohort. (A) Scatterplot showing a strong positive correlation between rDOI and pDOI (Spearman's *ρ* = 0.75, *p* < 0.001), with regression line and 95% confidence band. (B) Bland–Altman plot demonstrating a mean difference of 0.3 mm and limits of agreement from −4.6 to 5.2 mm, indicating no systematic measurement bias and expected variability at shallow invasion depths.

### 3.2 Predictive Value of DOI and DMI for VFM and Muscle Infiltration

The diagnostic performance of pDOI and rDOI in predicting altered VFM was first evaluated using ROC analysis. pDOI demonstrated moderate discriminative ability, with an AUC of 0.73, while rDOI yielded an AUC of 0.68, with no significant difference between the curves (DeLong *p* = 0.47), indicating comparable performance. The optimal pDOI cutoff for predicting altered VFM was 2.1 mm, corresponding to a sensitivity of 66% and a specificity of 73%, with a Youden index of 1.39.

On the other side, ROC analysis demonstrated that pDOI had excellent ability to predict vocal muscle infiltration, with an AUC of 0.94. The optimal cutoff was 2.2 mm (sensitivity of 88.4%). rDOI also showed strong performance for this endpoint, with an AUC of 0.88 and an optimal threshold of 3.2 mm (sensitivity of 84%). In addition to DOI‐based parameters, we assessed whether pDMI could predict altered VFM. ROC analysis yielded an AUC of 0.69, indicating fair discriminative capacity. The optimal pDMI cutoff was 0.9 mm (sensitivity of 44.8%) (Figure 4).

**FIGURE 4:**
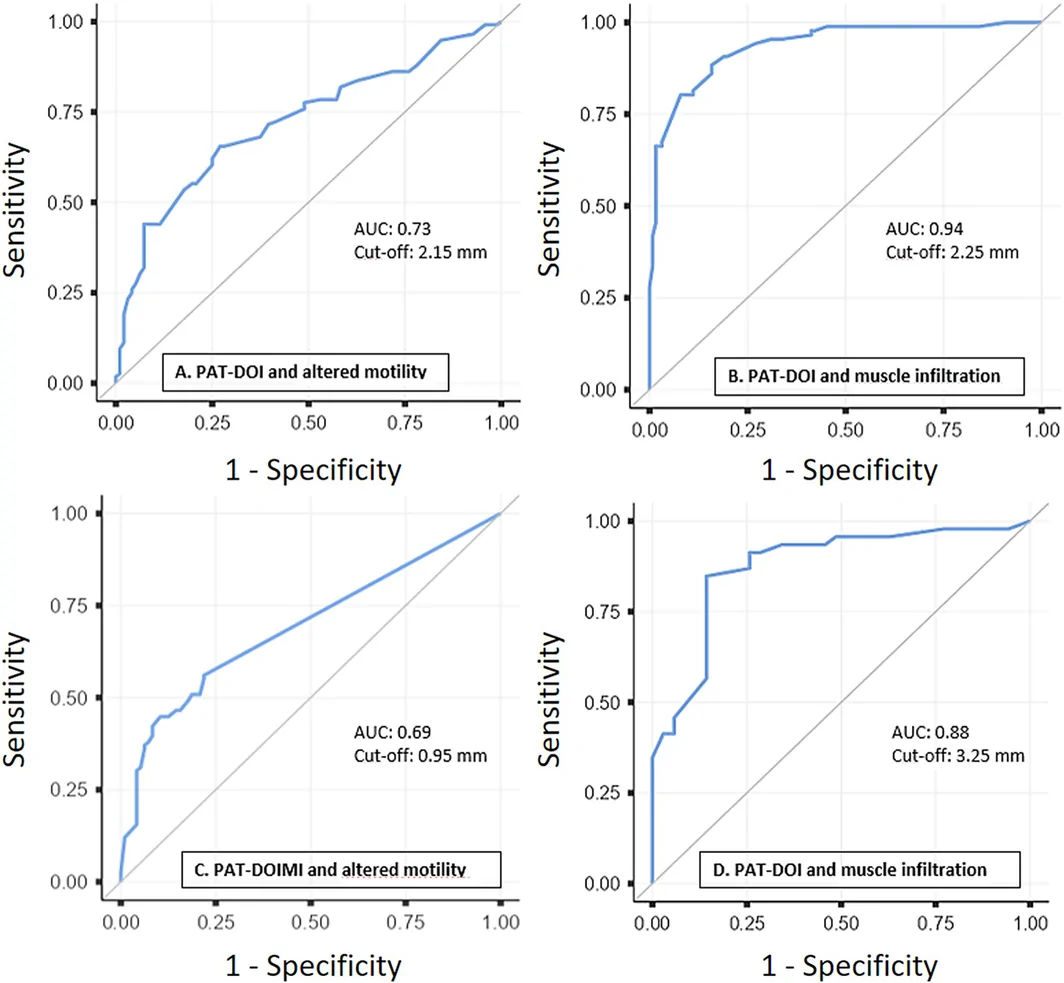
Receiver operating characteristic (ROC) curves and area under the curve (AUC) evaluating the diagnostic performance of depth of invasion (DOI) metrics in the derivation cohort. (A) Pathological DOI in predicting altered vocal fold motility (AUC = 0.73; optimal cutoff = 2.1 mm). (B) Pathological DOI in predicting deep vocal muscle infiltration (AUC = 0.94; cutoff = 2.2 mm). (C) Pathologic DMI in predicting altered vocal fold motility (AUC = 0.69; cutoff = 0.9 mm). (D) Radiologic DOI in predicting vocal muscle infiltration (AUC = 0.88; cutoff = 3.2 mm). Together, these ROC curves illustrate the strong diagnostic performance of DOI‐based parameters, particularly for identifying muscle invasion. [Color figure can be viewed in the online issue, which is available at www.laryngoscope.com]

### 3.3 Validation Analysis

In the IEO validation cohort, 86 patients were suitable for rDOI measurement, with 38 pT2–pT3 lesions treated with TOLMS having paired rDOI and pDOI for correlation, concordance, and Bland–Altman analyses. Interobserver agreement for endoscopic VFM assessment was excellent (agreement = 96%; Fleiss' *k* = 0.922). Radiologic–pathologic comparison showed a significant correlation between rDOI and pDOI (Spearman's *ρ* = 0.56, *p* < 0.001). In the 38 pT2–pT3 cases with paired measurements, Bland–Altman analysis revealed a mean bias of −0.2 mm (95% CI –0.8 to 0.3) and limits of agreement from −3.8 to +3.4 mm, indicating no systematic error. Lin's CCC confirmed moderate concordance (CCC = 0.56; 95% CI 0.30–0.75). Compared with the Genoa derivation cohort, the IEO cohort showed a lower but still significant rDOI–pDOI association, consistent with its wider pT distribution and greater dispersion on rDOI versus pDOI plots. Genoa cases clustered at low DOI (mostly early‐stage), yielding tighter linearity and stronger concordance, whereas IEO included more infiltrative pT2–pT3 tumors with broader DOI spread. Despite this, correlations remained highly significant (*p* < 0.001) with similar agreement patterns, supporting DOI as a robust, externally valid objective parameter in glottic SCC.

At ROC analysis, pDOI demonstrated excellent ability to discriminate normal from altered VFM (AUC = 0.870; 95% CI 0.816–0.924; *p* < 0.001). The IEO‐derived optimal threshold was 1.9 mm, yielding a sensitivity of 94.3%, a specificity of 77.3%, a PPV of 76.6%, an NPV of 94.4%, and an accuracy of 84.8%, confirming that even minimal DOI increases relate to functional impairment. pDOI was similarly effective in predicting DMI (AUC = 0.867; 95% CI 0.812–0.921; *p* < 0.001). The Youden‐optimal threshold (1.9 mm) provided a sensitivity of 93.1% (95% CI 85.6%–97.4%), a specificity of 76.4% (95% CI 67.3%–83.9%), a PPV of 75.7%, an NPV of 93.3%, and an overall accuracy of 83.8%. The corresponding likelihood ratios (LR+ = 3.94; LR− = 0.09) confirmed excellent rule‐in/rule‐out performance.

When applied to the IEO cohort, the Genoa‐derived DOI thresholds showed strong reproducibility. The 2.1 mm cut‐off for altered VFM performed robustly, with a sensitivity of 87.4% (95% CI 78.5%–93.5%), specificity of 82.7% (95% CI 74.4%–89.3%), and an overall accuracy of 84.8% (95% CI 79.0%–89.5%). Predictive values were favorable (PPV 80%, NPV 89.2%), and likelihood ratios (LR^+^ 5.06, LR^−^ 0.15) confirmed solid rule‐in/rule‐out capability. The 2.2 mm cut‐off for DMI showed comparable performance, with a sensitivity of 85.1% (95% CI 75.8%–91.8%), specificity of 83.6% (95% CI 75.4%–90%), and accuracy of 84.3% (95% CI 78.4%–89.1%). Its predictive values (PPV 80.4%, NPV 87.6%) and likelihood ratios (LR^+^ 5.20, LR^−^ 0.18) further supported its reliability.

Overall, both thresholds retained high discriminative accuracy in an independent, higher‐stage population, underscoring their cross‐institutional applicability.

Fagan nomograms were constructed on the observed prevalence of altered VFM (44.6%) and muscle infiltration (44.3%) observed in the IEO cohort (Figure 5). When applying the 2.1 mm cut‐off for predicting altered VFM, the positive likelihood ratio (PLR = 5.057) increased the post‐test probability from 44.6% to 80.3%, whereas the negative likelihood ratio (NLR = 0.1528) reduced it to 11% in case of a negative test. Similarly, a 2.2 mm threshold increased the post‐test probability of DMI from 44.3% to 80.5% (PLR = 5.198), and lowered it to 12.4% when negative (NLR = 0.1787).

**FIGURE 5:**
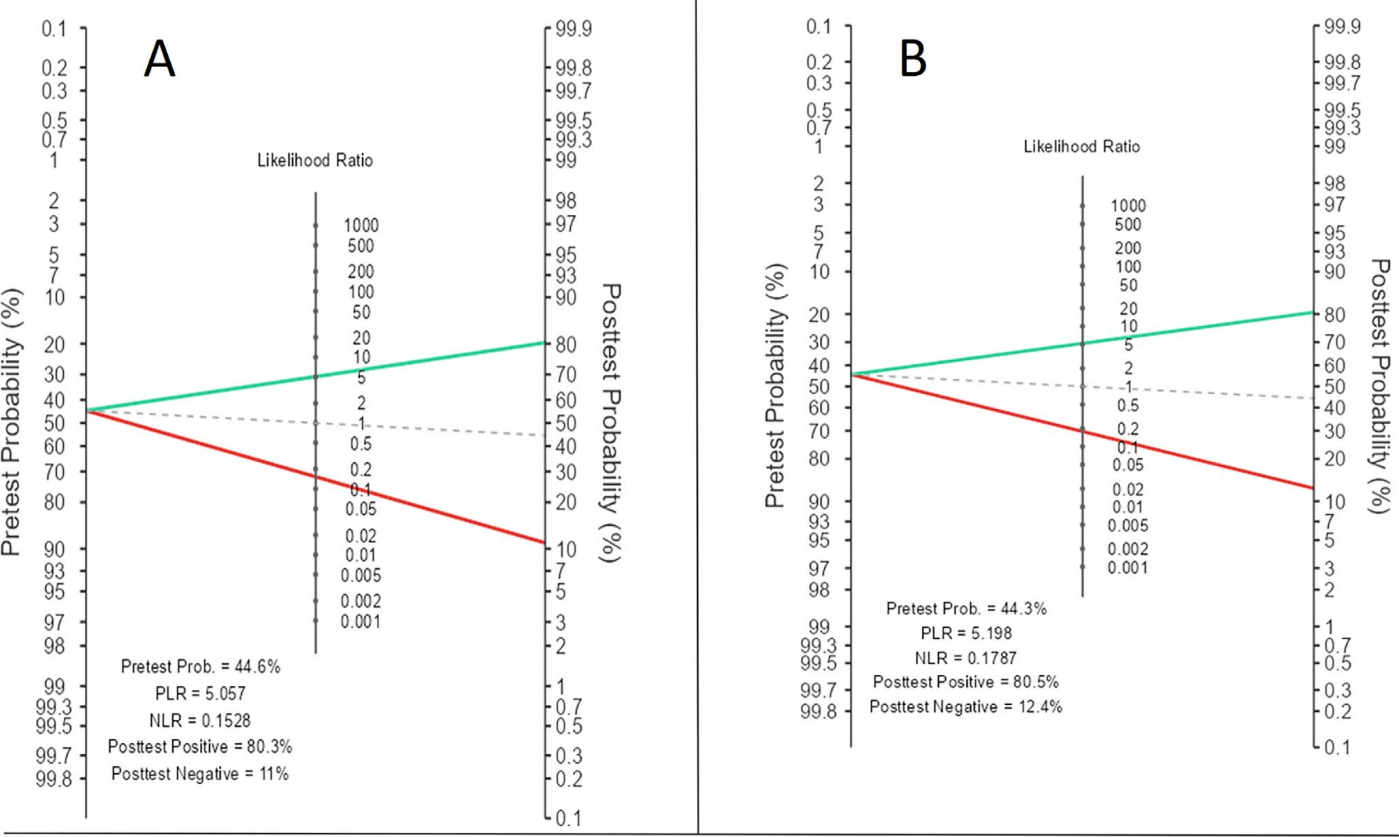
Fagan nomograms evaluating the diagnostic impact of the Genova‐derived depth of invasion (DOI) thresholds in the validation cohort. (A) For altered vocal fold motility, the 2.1 mm cutoff increases post‐test probability to 80.3% (Positive Likelihood Ratio—PLR = 5.06) and lowers it to approximately 11% (Negative Likelihood Ratio—NLR = 0.15). (B) For muscle infiltration, the 2.2 mm cutoff raises post‐test probability to 80.5% (PLR = 5.20) and reduces it to 12.4% (NLR = 0.18). [Color figure can be viewed in the online issue, which is available at www.laryngoscope.com]

## 4 Discussion

The butterfly effect, a concept originating from the chaos theory [10], highlights how small differences in initial conditions can lead to large, unpredictable outcomes. In laryngeal cancer staging, this underscores how subjective assessment of VFM can significantly impact treatment decisions and prognosis. A clinician's assessment of VFM can initiate a cascade of downstream consequences, mirroring the non‐linear dynamics observed in complex systems.

Relying on subjective staging criteria significantly affects therapy and prognosis. Differentiating cT2 (impaired motility) from cT3 (fixation) tumors directly influences treatment choices and limits less invasive options, ranging from exclusive RT to CRT for more advanced disease [11].

The lack of histopathological confirmation in non‐surgical cohorts complicates the interpretation of clinical and radiologic findings. Discrepancies between clinical and pathological staging are most pronounced in T2–T3 tumors, emphasizing the inherent limitations of subjective assessment [12, 13, 14].

Assessment is further confounded by factors like false vocal fold hypertrophy, lesion site, and mass effect, which can shift the perceived T category. Ferrari et al. [15] in a study involving 2170 endoscopic evaluations highlighted that assessment of VFM is highly subjective and its clinical relevance should be therefore reconsidered. These inconsistencies, observed even in high‐volume centers, suggest that interobserver variability is likely higher in less specialized institutions.

An objective, measurable pre‐treatment parameter could reduce subjectivity in VFM assessment, lessen clinical–pathologic staging discrepancies, and improve consistency in treatment decisions and prognostic accuracy.

Efforts to make this evaluation more objective began with Hirano in 1991 [16], who examined how vocal muscle invasion leads to impaired laryngeal motility. More recently, Filauro et al. [17, 18] demonstrated that DOI correlates with perineural, lymphovascular invasion, and nodal metastasis, and can be reliably assessed preoperatively using CT. Nevertheless, a limitation shared by these studies is the absence of a quantifiable parameter to determine the presence of vocal muscle infiltration.

Numerous studies in the oral cavity have underscored the significance of DOI and its radiological–pathological correlation. As a result, in the 8th edition of the TNM classification [1], DOI thresholds have been formally incorporated into the staging of oral cavity SCC [19, 20, 21]. Inspired by oral cavity studies, we investigated the relationship between DOI, VFM, and muscle infiltration. Due to the larynx's complex anatomy, glottic SCC was chosen to assess tumor extension from superficial layers to vocal muscle involvement.

Building upon this concept, we found a statistically significant correlation between rDOI and pDOI in both derivation and validation cohorts, supporting feasibility and reproducibility. These results suggest rDOI can be broadly applied when standardized protocols are followed, providing an objective measure for a traditionally subjective assessment.

Our findings indicate that increasing DOI is associated with greater pDMI, with strong discriminative performance on ROC analysis; pDOI value > 2.2 mm was statistically correlated with vocal muscle infiltration (AUC = 0.94), while a threshold of 2.1 mm was associated with impaired VFM, with lower accuracy, likely reflecting the limited interobserver agreement of VFM assessment (71%, improving to 86.3% after consensus).

Importantly, these results highlight the clinical relevance of radiologic evaluation even in tumors endoscopically classified as “early stage.” Whereas VFM represents a subjective and operator‐dependent parameter, rDOI provides a quantitative measure of tumor extension. Considering pDOI as the reference, Bland–Altman analyses demonstrated that rDOI closely approximates true infiltration depth, with minimal inter‐institutional bias (0.3 and −0.2 mm, respectively) and limits of agreement consistent with the intrinsic resolution of cross‐sectional imaging, supporting multicenter reproducibility.

When integrated with the validated pDOI cutoff for muscle infiltration (≈2 mm), rDOI near this range indicates a sharply rising risk of muscular invasion. Above 2.2 mm, post‐test probability rises to over 80%, while a negative test reduces it to ~12%, confirming strong rule‐in and rule‐out performance.

Although rDOI cannot directly visualize microscopic muscle invasion, its agreement with pDOI and its robust likelihood ratios make it a reliable preoperative proxy for identifying infiltrative glottic SCC. This radiologic information may be equally, if not more, valuable in non‐surgical patients, where pathology is unavailable and objective DOI estimates can improve risk stratification and guide treatment.

The literature provides variable reference values concerning the vocal muscle thickness: Tamaya et al. [22] reported an average of 4 mm; Hamdan et al. [23] described measurements between 5.3 and 5.5 mm; and Alonso et al. [24] reported 6.8 mm in males and 5.9 mm in females. This variability highlights the importance of accurate anatomical characterization in defining tumor extension and staging.

In this framework, rDOI in the 2 mm range does not merely correlate with altered vocal fold mobility—it quantitatively strengthens the clinician's initial suspicion, providing a reproducible, morphologic parameter that bridges endoscopic observation and histopathological confirmation. From a surgical planning perspective, rDOI values in the 2 mm range may alert the surgeon to a higher probability of vocal muscle involvement and may support consideration of more extensive cordectomy according to the European Laryngological Society classification [25]. However, the present study was not designed to establish DOI‐based treatment rules, and surgical decisions should continue to integrate endoscopic findings, lesion location, exposure, patient factors, and surgeon judgment.

Furthermore, in an era in which medicine is increasingly moving toward the integration of artificial intelligence, the availability of objective data could positively contribute to training algorithms.

This study has inherent limitations. Its retrospective design and institutional differences, with different modalities of surgical approach, may have introduced variability in radiologic and pathologic measurements. The predominance of early‐stage tumors in the derivation cohort also led to zero‐inflated DMI distributions, which may have influenced radiologic–pathologic concordance. In addition, although both CT and MRI were used for preoperative assessment, the study was not powered or specifically designed to compare the diagnostic performance of these imaging modalities. Therefore, no recommendation can be made regarding the preferential use of CT versus MRI for DOI assessment, and dedicated prospective, modality‐specific studies are needed. Another limitation is that lesion location along the vocal fold was not systematically analyzed. Tumors involving the anterior, middle, or posterior glottis may differ in their relationship with the vocal ligament, vocal muscle, and paraglottic space, and these anatomic differences may influence the clinical impact of invasion on vocal fold motility. Future studies should incorporate lesion topography to determine whether DOI thresholds vary according to glottic subsite. Finally, treatment‐related factors were not analyzed as outcome modifiers. In the derivation cohort, patients underwent different surgical approaches, including TOLMS, open partial laryngectomy, and total laryngectomy, whereas the validation cohort included patients treated uniformly with TOLMS. Moreover, the present study was not designed to evaluate whether DOI‐guided risk stratification modifies treatment selection or improves functional, oncologic, or survival outcomes. The present work should therefore be viewed as a foundational step toward future prospective multicenter studies evaluating whether infiltration depth carries prognostic significance in glottic SCC and whether it can be incorporated into staging or treatment algorithms.

## 5 Conclusion

The study shows that depth of invasion is a reliable, objective, and reproducible morphologic parameter in glottic SCC, addressing limitations of subjective VFM assessment. rDOI correlated well with pDOI, serving as a valid preoperative surrogate of tumor infiltration. Thresholds around 2–2.2 mm showed promising associations with vocal fold functional impairment and muscle invasion; however, these findings should be interpreted in light of the study's retrospective design and limited ability to compare CT and MRI, assess lesion‐location effects, or evaluate treatment‐related outcomes. Prospective multicenter studies are needed to determine whether DOI can improve staging consistency, guide treatment selection, or provide prognostic information beyond current clinical assessment.

## Funding

The authors have nothing to report.

## Conflicts of Interest

The authors declare no conflicts of interest.

## Data Availability

The data that support the findings of this study are available on request from the corresponding author. The data are not publicly available due to privacy or ethical restrictions.
